# Report of Cases

**Published:** 1873-06

**Authors:** W. D. Hoyt

**Affiliations:** Rome, Georgia, Member of Committee on Practice of Medicine for the Seventh Congressional District


### REPORT OF CASES,

BY W. D. HOYT, M.D., ROME, GEORGIA,

*Member of Committee on Practice of Medicine for the Seventh Congressional District.*

Not being the Chairman of the Committee on the Practice of Medicine for the Seventh Congressional District, but simply a member of it, it will, perhaps, be considered a work of supererogation to make a report at all.

It will clearly be impossible for me to give more than a slight sketch of the various diseases we have, and it is obviously necessary, as well as becomingly modest, to confine even this sketch to my own little corner of the District, leaving the general report to the chairman and our other Atlanta associate. I propose, therefore, after glancing briefly at the epidemics and prevalent diseases in this locality, to report more fully two cases —one of typho-malarial fever, and the other of malarial engorgement of the lung—believing that there are points of interest in the history, treatment and termination of these cases, at least to practitioners in a similar locality.

With the exception of the all-pervading epidemic and epizootic influenza, which visited us in common with other portions of the country, we have been singularly free from epidemics during the past twelve months. The influenza presented the same variety in its manifestations, and in the organ attacked, here that it did elsewhere; but I judge, from the fact that I have heard of no deaths from meningitis since its advent, that the attacks on the brain were less severe and more amenable to treatment here than in some other localities. This would seem to indicate that the idea that there is in some localities at times special additional influence determining to the brain, is a correct one. I judge that the other manifestations of the disease were no less violent and universal here than elsewhere.

Scarlet fever has on two occasions during the last two years made its appearance amongst us, but has not spread; not more than four or five persons were affected at each of its visitations. This fact, coupled with the light returns of deaths from this malady at the South, in the census mortuary reports, would seem to indicate that the South is not a favorable soil for the growth of this terrible disease. I certainly have not seen such cases of it during the seven years I have practiced medicine at the South, as I saw during a like term of practice in Philadelphia.

Pneumonia, dysentery and consumption have, of course, levied their usual toll upon our population. Not having practiced medicine at the South before the war, I am not as well prepared to judge of the increase of the latter disease among the negroes as others with whom I have conversed. From what I learn from their testimony, I judge that the changed habits of the race have greatly increased the frequency of the disease amongst them. Certain it is, that its victims amongst the colored people are numerous, and that the disease in its progress is often frightfully rapid.

Malarial diseases have not been unusually prevalent here, except in certain localities where local influences intensified its action. I saw one case of malarial hæmaturia in consultation. The patient lived on the banks of a creek about eight miles from Rome, which creek had been dammed to afford water power. The patient after several paroxysms recovered. This is the only case of the kind I have seen in this region, though other cases occurred in the same neighborhood.

Whether it is that our being situated in a region intermediate between the malarial district south of us and the regions of typhoid fever above us, the typhoid and malarial elements of disease are here blended, and occasionally both affect the same individual at the same time; or whether the malarial element, being always present, modifies the typhoid element when it occasionally crops out; certain it is that we occasionally encounter here cases of typho-malarial fever, and that cases of pure typhoid fever are rather rare. These cases of typho-malarial fever when severe are very unsatisfactory to treat; the disease seeming to borrow intensity from the malarial element and persistency from the typhoid element. Two of these cases well marked—one of which I saw with Dr. Battey, and the other he saw with me—both terminated fatally. This was in the year '66 or '67. The patients were worn out with fevers, which, though markedly paroxysmal, quinine in the doses used did not control; it was, therefore, with gloomy forebodings that in the following case I diagnosed

*Typho-Malarial Fever—Recovery Under Large Doses of Quinine.*

Case I.—On the 26th of September I was called to see Mr. S., a stout Pennsylvanian, about twenty-one years of age, somewhat pitted with the small pox, who had been employed in our town for a few months. He had been ailing about a week before employing a physician. I was at first doubtful about the diagnosis; doubtful as to whether I had on hand a case of malarial disease, which would yield readily to treatment, or a case of typhoid, which would drag its slow length along. Determining to give him the benefit of the doubt, I adopted an anti-malarial treatment, which I continued until the 28th, when on my visit I found him sitting in the common room of the hotel, dressed and feeling much better. On the 29th, I was called again to see him at the house of a friend, to which he had walked. He was not so well, and about 10 P.M. had quite a severe chill; at 10 A.M. of the 30th he had another, and at 10 P.M. another.

Determining to endeavor to remove the malarial element of his disease, I gave him, in anticipation of a chill on the 1st of October, fifteen grains of quinine. On the evening of that day Dr. C. H. Gorman saw him with me, and our prognosis of the case was gloomy. The pulse was 110, very feeble indeed, and quick. There had been slight bleeding from the nose. There was the characteristic stupor of typhoid; the tongue inclined to be dry, and the bowels rather loose. The symptoms, altogether, so early in the disease were far from encouraging. I gave him fifteen grains more of quinine.

Great was my delight on the morning of October 2d, to find him much improved. He had missed his chills; his pulse was at eighty, soft, regular and with more tone in it.

About 2 P.M. I was called again, and found that by a mistake of his nurse the quinine I had directed had not been given, and he had a chill for over an hour. I applied mustard down the spine and gave a dose of morphine, and soon afterwards reaction came on. There were no more mistakes about the quinine, and it was given for days in quantities of thirty grains a day—fifteen to ward off the morning paroxysms, fifteen against the evening. The other medication consisted in the use of turpentine emulsion and laudanum and whisky. No more chills came on, and the bowels were kept confined for about a week. He then complained of pain in the region of the transverse colon, and scybalæ could be felt there, for which copious enemeta were used. After using the quinine in quantities of thirty grains a day long enough to feel that the malarial element of the disease was well under control, the quantity was reduced, and afterwards left off, except to guard against hebdomadal paroxysms.

The malarial element having been removed from the case, it progressed as a moderately severe case of typhoid, the pulse ranging for perhaps two weeks at about ninety to one hundred, the stupor well marked, and sleeplessness being much complained of.

By the 14th he seemed convalescent, but being restless and having imprudently gone to the house of another friend, he had a relapse of typhoid, without any great admixture of the malarial element. For some time he had obstinate night sweats, and there being a point of dullness in the upper part of the right lung, with some cough, it was feared he might slip off into phthisis. Some cod liver oil, with iodide of iron, and an increase of his toddy, with good diet, at length built him up, and I ceased attending him on the 1st of November. He continued to convalesce, and in perhaps another month was ready for work.

The point of interest in this case is the effect on it of the large doses of quinine given. The practice of the German physicians seems to show that quinine is not as injurious in pure typhoid fever as we have been apt to suppose, and as previously given, at one period of the day only (in three doses), in cases of typho-malarial fever, the treatment produced no favorable results. I was led in this case to give it in twice the usual quantity, in consequence of the double paroxysms, with I believe the best results. I shall, at least, not hesitate to repeat the treatment should a similar case present itself.

*Malarial Engorgement of the Lung—Treatment by Large Doses of Quinine—Recovery.*

CASE II.—I was called January 13th, 1873, to see C. L. O., aged nineteen years ; a healthy young man, of a florid complexion and sanguine temperament.

He had been attacked the day before with a chill and pain in the left lung, having previously suffered for some time with a cold. I found him with a pulse of one hundred and twenty, irritable and gaseous, but weak. He had a peculiar thick white coat, almost like cotton. The expectoration was difficult, and the sputa bloody. Applied two wet and several dry cups; gave veratrum with the view of preventing as far as possible a further extension of the disease; gave a calomel purge; applied a jacket poultice, and directed fifteen grains of quinine to be given between 2 and 6 A.M.

14th.—He was easier. The skin was cool and perspiring; pulse one hundred; tongue cleaning from the tips and edges. Gave small doses of calomel and quinine; continued the poultice, and directed quinine next morning.

15th.—Condition much the same; tongue still cleaning. There was still pain over left lung, which revealed bronchial respiration on auscultation, and dullness on percussion. Applied a blister; continued calomel and Dover's powder; quinine to be repeated.

16th.—Pain less severe; tongue quite clean; lung apparently cleaning up. The disease has not advanced, the area of dullness not extending above the base of the scapula. Discontinued the quinine, calomel and Dover's; gave an expectorant mixture and continued poultice.

17th.—Crepitation abundant throughout the dull portion of lung. Every indication of recovery.

18th.—Not so well; pulse above one hundred; tongue showing a dry stripe down the center. Some mutterings and wandering of the mind as he sleeps, which he does most of the time; not quite himself always when awake; symptoms decidedly typhoid in their character. Twice in the twenty-four hours, about noon and midnight, there is a deep-red spot on the left cheek, which gradually fades out. Expectoration more difficult, and sputa more bloody than for two or three days. It seemed to me, at my evening visit, that the dullness was more complete and extensive than at my morning visit. Gave turpentine emulsion and carbonate of ammonia, instead of the chlorate of potassa mixture I had been using.

19th.—Symptoms still worse. There is a decided increase of typhoid symptoms—picking at the bedclothes and muttering; his mind wandering much of the time, but he can be aroused; the tongue is still dryer, and the pulse one hundred and ten. If some change for the better does not occur, he cannot live more than forty-eight hours.

Was I mistaken in thinking that the dullness in the lung was more complete the evening before than it had been? Certainly there was now crepitation throughout the lung, but the area of dullness was much greater than two days before, extending nearly to the apex.

On repeating my visit at noon, I found the painted spot on the left cheek. There was a central spot unduly white, surrounded by a deep-red areola. The amount and degree of dullness in the lung was increased since morning. The amount of blood in the sputa is greater; it is fresher in color. I could not imagine an inflammation varying in intensity in this way. Had it been a veritable pneumonia, it should have progressed to recovery after the returning crepitation manifested itself. I said to myself: "What I see on the cheek is a picture of what is going on in the lung. There is twice in the twenty-four hours a disturbance in the circulation of the lung, just as there is in the cheek. There is a contraction of the capillaries, followed by dilatation and accompanied by engorgement of the surrounding tissue, thus constantly extending the area of the dullness. This disturbance is due to a malarial cause. To combat this malarial cause of disturbance, I must give a specific remedy — quinine." Discontinuing all other medicines, I accordingly gave him fifteen grains of quinine, in anticipation of the night paroxysm. The patient after getting under its effects rested more quietly, and with less cough than before, and under the effects of a small dose of calomel slept well. There was the slightest possible symptom of a paroxysm. To take fifteen grains of quinine in the morning.

20th.—Patient better; tongue moistening; pulse one hundred. He has very little cough. Lungs freely crepitant throughout. Continue quinine; thirty grains in twenty-four hours. Continue jacket poultice.

21st.—Patient nearly well; pulse seventy-six, of good volume; tongue perfectly moist. He has scarcely any cough, and only slight mucous expectoration; lungs resonant throughout, and only a few loose mucous rales to be heard. Continue quinine.

22d.—Patient still improving; pulse seventy-two; tongue moist and clean; lung almost normal. Thanks to quinine, the patient passed with astonishing rapidity from the brink of the grave to convalescence and health. Directing the remedy to be given in such a way as to guard against fresh paroxysms, I ceased attendance on him.

I called this case one of malarial engorgement of the lung, and such I imagine it to have been. Had there been inflammation producing an effusion of lymph into the lung and causing the extensive dullness found, its products couid not have been absorbed or gotten rid of in any other way in three days. The lung was really engorged with blood from a malarial disturbance in the circulation, and quinine stopped the paroxysms, and at the same time restoring tone to the blood vessels, emptied them of their surplus blood, thus removing the traces of the disease.

A few words with reference to the differential diagnosis between this disease and pneumonia, with which it may readily be confounded, may not be out of place. The dullness and crepitation in the two diseases are, I think, not distinguishable from each other. The sputa are, if I mistake not, less rusty-colored and more florid than in pneumonia. The chief point, however, in differentiating the two diseases, is the variation in the condition of the lung in engorgement. Instead of progressing steadily to increasing and uniform dullness, and then in the progress of the case presenting returning crepitation, to be followed by mucous rales as in convalescing pneumonia, there will be a variable amount of dullness at different periods of the day—the dullness increasing and diminishing as the tide of engorgement ebbs and flows. It is of the utmost importance to success in treatment to distinguish between the two diseases. No amount of antiphlogistic treatment can prevent fresh engorgements of the lung, or do more than temporarily relieve them. The disease is due to a specific cause, and must have a specific treatment.

---

The late eminent Dr. Louis, of Paris, left a legacy to the Medical Association of the Seine, which will add over three hundred dollars a year to the income of that body. It is strange, certainly, that bequests of this kind are so rare, since they would in many instances be greatly to the advancement of science.

